# Extracellular vesicles, syntaxin 2 and SNAP23 in the uterine microenvironment of the rat

**DOI:** 10.1530/REP-24-0188

**Published:** 2025-05-29

**Authors:** Sadaf N Kalam, Samson N Dowland, Louise Cole, Laura A Lindsay, Christopher R Murphy

**Affiliations:** ^1^ School of Medical Sciences, Faculty of Medicine and Health, The University of Sydney, Sydney, New South Wales, Australia; ^2^ Australian Institute for Microbiology and Infection, The Ithree Institute, Faculty of Science, University of Technology Sydney, Sydney, New South Wales, Australia

**Keywords:** receptivity, SNAP23, uterine luminal fluid, pregnancy, extracellular vesicles

## Abstract

**In brief:**

During uterine receptivity, exocytosis from uterine epithelial cells (UECs) contributes to uterine fluid composition, playing a role in communication with an implanting blastocyst. SNAREs, a family of proteins involved in exocytosis, are increased in the receptive uterine epithelium of the rat.

**Abstract:**

Uterine luminal fluid is composed of secretions from the uterine luminal and glandular epithelial cells. The fluid composition plays a role in cell-to-cell communication between the receptive endometrium and an invading blastocyst. Part of this fluid is released from the epithelial cells via exocytosis, mechanisms regulating this are not yet understood. Using transmission electron microscopy, this study identified extracellular vesicles in the uterine lumen at the time of fertilisation and uterine receptivity. Immunofluorescence microscopy showed SNARE proteins syntaxin 2 and SNAP23 in the apical area of UECs at the time of receptivity. SNAP23 was also found in the uterine fluid on day 5.5 of early pregnancy. Western blotting of isolated UECs demonstrated a significant increase in syntaxin 2 and SNAP23 at the time of uterine receptivity compared to the time of fertilisation. The morphological evidence of extracellular vesicles in the uterine lumen and the presence of SNARE proteins syntaxin 2 and SNAP23 in the apical part of the luminal epithelium at the time of uterine receptivity suggests that exocytosis contributes to the composition of the uterine luminal fluid, a potential component of maternal–foetal communication during early pregnancy.

## Introduction

Blastocyst implantation in the uterus involves coordinated dialogue between the blastocyst and a receptive endometrium (Carson *et al.* 2000, Lessey 2000, Murphy 2004). The endometrium and specifically the luminal uterine epithelial cells (UECs) undergo defined morphological changes to establish uterine receptivity. Some changes to UECs during uterine receptivity include the loss of regular microvilli and non-adhesive proteins from the apical plasma membrane and reorganisation of microtubules and the actin terminal web (Aplin 1997, Murphy 2004, Moore *et al.* 2016, Kalam *et al.* 2018). In addition, the UECs apical surface displays adhesive proteins, pinopods, an increase in plasma membrane cholesterol and porosomes (Murphy & Martin 1985, Murphy & Dwarte 1987, Murphy 1993, 1994, 2004, Singh & Aplin 2009, Kalam *et al.* 2022). There are also changes in the volume and fluid content of the luminal microenvironment that play a role in blastocyst implantation (Glasser *et al.* 1987, Erranz *et al.* 2004, Aghajanova *et al.* 2008, Ng *et al.* 2013, Salamonsen *et al.* 2016).

Uterine luminal fluid (ULF), once considered simply the medium for sperm and embryo transport, is now thought to play a major part in co-ordinating cell-to-cell communication between a receptive endometrium and an invading blastocyst (Zhang *et al.* 2017), with significant changes in composition during the reproductive cycle and peri-implantation period (Salleh *et al.* 2005, Kelleher *et al.* 2016). The protein composition of ULF may play a direct role in human infertility as there were significant changes in secretory proteins found in ULF of fertile compared to infertile women (Fitzgerald *et al.* 2018).

Extracellular vesicles (EVs) are membrane surrounded vesicles secreted by all cell types carrying a range of cargo and have been detected in ULF of a number of species including sheep, cattle, mice, rats and human, where the uterus is thought to be one of the possible source of these EVs (Griffiths *et al.* 2008, Burns *et al.* 2014, Tannetta *et al.* 2014, Zhang *et al.* 2017, Nakahara *et al.* 2020, Hart *et al.* 2022, Paul *et al.* 2023, Piibor *et al.* 2023, Zierden *et al.* 2023, Huang *et al.* 2024). In cell culture, EVs have been reported to be secreted by uterine endometrial cancer cells (Ng *et al.* 2013, Franchi *et al.* 2016, Nguyen *et al.* 2016). These EVs contained in ULF are thought to contain a variety of regulatory molecules such as microRNAs, proteins and lipids that may mediate intercellular communication between maternal and foetal tissue (Nguyen *et al.* 2016, Tan *et al.* 2020).

Intracellular vesicles are seen morphologically in the apical cytoplasm of receptive rat UECs peaking on day 5 of early pregnancy and then disappearing after uterine receptivity (Parr 1982). These vesicles could fuse with the apical membrane, incorporating receptive proteins into the apical plasma membrane or could be released into the uterine lumen, contributing to the composition of ULF and playing a role in maternal–foetal communication and blastocyst nourishment. Mechanisms of exocytosis may play a role in release of these cytoplasmic vesicles.

Soluble NSF attachment proteins (SNAPs) and SNAP receptor (SNARE) proteins are a large superfamily of proteins that mediate vesicle fusion. The SNARE core complex requires a vesicle SNARE (v-SNARE) such as vesicle-associated membrane protein (VAMP) located on a vesicle membrane and two target SNAREs (t-SNARE), a syntaxin and a SNAP23/25, located in the target plasma membrane (Söllner *et al.* 1993, Sutton *et al.* 1998). T-SNAREs syntaxin 2 and SNAP23 have been shown to control apical secretions from polarised epithelia of lung and pancreas (Imai *et al.* 2003, Abonyo *et al.* 2004). Previous studies have shown a significant increase in the v-SNARE VAMP2 in rat UECs at the time of uterine receptivity compared to fertilisation (Kalam *et al.* 2020). Due to the role of syntaxin 2 and SNAP23 in luminal secretions in other secretory organs and the appearance of VAMP2 in rat UECs during uterine receptivity, the present study will investigate the localisation and distribution of these proteins in UECs during early pregnancy.

## Materials and methods

### Animals and mating

This study used female virgin Wistar rats aged 10–12 weeks and all procedures were approved by The University of Sydney Animal Ethics Committee. Rats were housed in plastic cages at 21°C under a 12 h light:12 h darkness cycle and were provided with free access to food and water. Pro-oestrus female rats were mated overnight with males of proven fertility. The presence of sperm in a vaginal smear the following morning (approximately 09:00 h) indicated successful mating, and this was designated day 1 of pregnancy. Rats designated for half day pregnancy time point such as day 3.5 and 5.5 were sacrificed at approximately 15:30 h. Uterine tissues were collected from five rats each on days 1 (time of fertilisation), 3.5 (midpoint between fertilisation and receptivity), 5.5 (time of receptivity-apposition), 6 (time of receptivity-adhesion) and 7 (tail end of time of receptivity-penetration and attachment) of pregnancy for immunofluorescence and western blot analysis. Further uterine tissue was collected from four rats each on days 1, 5.5 and 6 for transmission electron microscopy (TEM).

Rats were administered 20 mg/kg of sodium pentobarbitone (Vibac Animal Health, Australia) intraperitoneally and the uterine horns were collected under deep anaesthesia, before euthanasia. The uterine horns were then used for immunofluorescence microscopy, western blotting, and TEM.

### Transmission electron microscopy

Uteri were cut into 5 mm pieces and fixed in Karnovsky’s fixative (2.5% glutaraldehyde (ProSciTech, Australia), 2% paraformaldehyde (ProSciTech) in 0.1 M Sorenson’s phosphate buffer (PB, pH 7.4)) for 45 min. All processing took place at room temperature (RT), unless otherwise stated. The tissue was further cut into 0.5–1 mm slices under a droplet of fixative and returned to fresh fixative for another 45 min. The tissue was washed twice for 5 min in 0.1 M PB and then washed again with 0.1 M maleate buffer (MB) twice for 5 min. Next, the tissue was post-fixed with 1% tannic acid in 0.1 M MB for 40 min. Tissue was rinsed in 0.1 M MB twice for 5 min, then dH_2_O twice for 5 min and further fixed with 1% uranyl acetate in dH_2_O for 1 h. Tissue was rinsed again in dH_2_O three times for 5 min and dehydrated with a graded series of ethanol and then infiltrated with Spurr’s resin (SPI Supplies, England, UK). Samples were embedded in fresh Spurr’s resin in BEEM^®^ capsules (ProSciTech) and polymerised at 60°C for 24 h. Two blocks per animal were cut using a Leica Ultracut S ultramicrotome and sections 60–70 nm thick were mounted onto 400-mesh copper grids. Sections were post-stained with 2% uranyl acetate in dH_2_O for 10 min and then with Reynold’s lead citrate for 10 min. Sections were examined in a Jeol 1400 TEM (Jeol Ltd, Japan) at 100 kV.

### Immunofluorescence microscopy of syntaxin 2 and SNAP23

Uterine horns (5 mm pieces) were coated with OCT (Tissue Tek, USA), snap frozen in supercooled isopentane, and stored under liquid nitrogen until use. Frozen sections (7 μm) were cut using a Leica CM 3050 cryostat (Leica, Switzerland) and air-dried onto gelatine-chrome alum-coated slides.

Sections for syntaxin 2 staining were fixed in 4% formaldehyde in 0.1 M phosphate buffer, while those for SNAP23 were fixed in 75% methanol in phosphate buffered saline (PBS) for 10 min at RT. All sections were subsequently washed with PBS and blocked with 1% bovine serum albumin (BSA; Sigma Aldrich, USA) in PBS for 30 min at RT. Sections were incubated overnight at 4°C with rabbit monoclonal anti-syntaxin 2 antibody (7.8 μg/mL; Abcam, UK: ab170852) or goat polyclonal anti-SNAP23 antibody (1.25 μg/mL; Abcam, ab166808), diluted in 1% PBS/BSA. Concurrently, control sections were incubated with non-immune mouse or goat IgG (Sigma Aldrich) at the same concentration as the primary antibody. Sections were washed in PBS and incubated with FITC-conjugated goat anti-mouse IgG (3 μg/mL; Jackson ImmunoResearch, USA) or Alexa Fluor 488-conjugated donkey anti-goat IgG (0.02 μg/mL; Life Technologies Australia Pty Ltd, Australia) for 30 min at RT. Sections were washed in PBS and mounted onto glass slides with Vectashield-containing DAPI (Vector Laboratories, USA) and coverslipped (No. 1 thickness).

The Zeiss AxioImager M2 microscope (Carl Zeiss, Australia) was used to image the sections and images were acquired with the use of Zeiss AxioCam HR digital monochrome CCD camera (Carl Zeiss) and ZEN 2013 (Blue edition) software (Carl Zeiss).

The camera exposure time was set on the brightest sample per protein and the same parameters were used to image all other samples. These images were then used to quantify the fluorescence intensity.

### Image analysis

Intensity measurements were made using the FIJI (Fiji is just ImageJ). UECs on day 1 and 5.5 of early pregnancy were selected for intensity measurements and other areas were excluded from the region of analysis. Day 1 intensity measurement representative of non-receptive UECs were compared with day 5.5 showing receptive UECs. Cell counts were obtained from the DAPI channel by thresholding the nuclei and a particle size limit was set to eliminate any peripherally cut cells in the section. The FITC or Alexa 488 channel with the protein staining was thresholded at the same range for all images and a grey scale value was obtained for each image. These were then standardised to the number of nuclei to obtain a measurement of fluorescence intensity per cell. Three randomly selected high magnification images per rat were analysed and averaged to provide an average intensity per set. Unpaired two-tailed Student’s *t*-test analysis was performed and *P* < 0.05 was determined to be significant. All graphs were generated using the GraphPad Prism software (Version 7.02, GraphPad Software, Inc., USA) and error bars represent the mean ± SEM.

### Co-localisation analysis

For co-localisation analysis of SNAP23 and phalloidin staining, frozen sections of day 5.5 pregnant uteri were fixed in 50/50 absolute methanol/PFA for 10 min at RT. Then, sections were washed with PBS and blocked with 1% BSA (Sigma Aldrich) in PBS for 30 min at RT. Sections were then incubated overnight at 4°C with goat polyclonal SNAP23 (1.25 μg/mL; Abcam, ab166808) and diluted in 1% PBS/BSA. Sections were washed in PBS and incubated with the Alexa Fluor 488 donkey anti-goat IgG (0.02 μg/mL; Life Technologies) for 30 min at RT. Concurrently, control sections were incubated with non-immune IgG (Sigma Aldrich) at the same concentration as primary antibody. Sections were washed in PBS and then incubated with tetramethylrhodamine isothiocyanate (TRITC)-conjugated phalloidin (Sigma-Aldrich) diluted to 0.5 μg/mL in 1% BSA/PBS for 1 h to stain filamentous actin (F-actin). Sections were then washed with PBS and mounted with Vectashield with DAPI (Vector Laboratories) and coverslipped (No. 1 thickness). Alongside, SNAP23-only and phalloidin-only sections were prepared as controls. Confocal images were taken using a Zeiss LSM 800 confocal microscope (Carl Zeiss). Pearson co-localisation coefficient (PCC) was calculated using the co-localisation module in the ZEN 2 software (Carl Zeiss) and reported as an average from all animals. PCC close to 1 indicates protein co-localisation.

### Isolation of rat uterine luminal epithelial cells

UECs were isolated as previously described (Kaneko *et al.* 2008) and immediately placed into lysis buffer (50 mM Tris–HCl, pH 7.5, 1 mM EDTA, 150 mM NaCl, 0.1% SDS, 0.5% Deoxycholic acid, 1% Igepal and 1% protease inhibitor cocktail; Sigma Mammalian Cell lysis kit, Sigma Aldrich) with 10% PhosSTOP phosphatase inhibitor (Roche, Australia). The isolated cells were homogenised using a 1 mL syringe (Livingstone International, Australia) and centrifuged at 8,000 ***g*** at 4°C for 3 min. The supernatant was collected and frozen immediately in liquid nitrogen and stored at −80°C until use for western blotting.

### Western blot analysis

Protein concentrations were determined using the BCA protein assay (Micro BCA^TM^ protein assay kit; Thermo Fisher Scientific, USA) and CLARIOstar microplate reader (BMG labtech Durham, USA), according to the manufacturer’s instructions. For detecting syntaxin 2, protein samples (20 μg) and sample buffer (8% glycerol, 50 mM Tris–HCl, pH 6.8, 1.6% SDS, 0.024% bromophenol blue, 4% dithiotheitol (DTT)) were heated at 95°C for 10 min before loading. For detecting SNAP23, protein samples (20 μg) and sample buffer (8% glycerol, 50 mM Tris–HCl, pH 6.8, 1.6% SDS, 0.024% bromophenol blue, 4% β-mercaptoethanol) were heated at 95°C for 5 min before loading. Protein was separated on 12% FastCast SDS-polyacrylamide gel (Bio-Rad Laboratories, USA) through electrophoresis at 200 V for 40 min and transferred to a polyvinylidene dilfluoride (PVDF) membrane (Immunobilon™ transfer membrane; Millipore, USA) at 100 V for 90 min. Membranes stained with syntaxin 2 were blocked in 1% skim milk in TBS-t (10 mM Tris–HCl, pH 7.4, 150 mM NaCl, 0.05% Tween 20), whilst those stained with SNAP23 were blocked in 5% skim milk for 1 h RT with constant agitation. Membranes were then incubated with syntaxin 2 (1.95 μg/mL, Abcam, ab170852) or anti-SNAP23 (0.25 μg/mL, Abcam, ab166808) primary antibodies diluted in 1% skim milk in TBS-t overnight at 4°C on a rocking platform. The membranes were washed in TBS-t and subsequently incubated with goat anti-rabbit IgG horseradish peroxidase-conjugated secondary antibody (0.5 μg/mL; Dako, Australia) or rabbit anti-Goat IgG horseradish peroxidase-conjugated secondary antibody (0.125 μg/mL; Dako) for 2 h at RT with constant agitation. Protein bands were detected with Immobilon Western HRP Substrate (Merck Millipore) and images captured with a CCD camera and the Bio-Rad ChemiDoc MP System (Bio-Rad). Membranes were then incubated in stripping buffer (62.5 mM Tris–HCl (pH 6.7), 2% SDS and 100 mM β-mercaptoethanol) at 60°C for 45 min and reprobed with mouse monoclonal anti-β-actin antibody (0.4 μg/mL; Sigma Aldrich) overnight at 4°C and HRP-conjugated goat anti-mouse IgG (0.2 μg/mL; GE Healthcare, Australia) for 2 h at RT to ensure equal loading.

### Densitometry analysis

Protein band intensities were quantified with the Bio-Rad Image Lab 4.0 software (Bio-Rad) using the volume analysis tool with local background subtraction and normalised to β-actin band intensities from the same lane. Statistical analysis was performed on normalised intensities with the GraphPad Prism software (Version 7.02, GraphPad Software). Changes in quantity from day 1, 3.5, 5.5, 6 and 7 were analysed using ordinary one-way ANOVA. Five blots, with each blot containing day 1, 3.5, 5.5, 6 and 7, were used for statistical analysis. Representative blots are shown in Fig. 5. For blot transparency, uncropped blots are shown in Supplementary Fig. 1 (see section on Supplementary materials given at the end of the article). For multiple comparisons, Tukey’s post-hoc test was applied (reporting multiplicity-adjusted *P*-values) to determine which pairs of means were significantly different.

## Results

### EVs are present in the uterine lumen during early pregnancy

**Figure 1:**
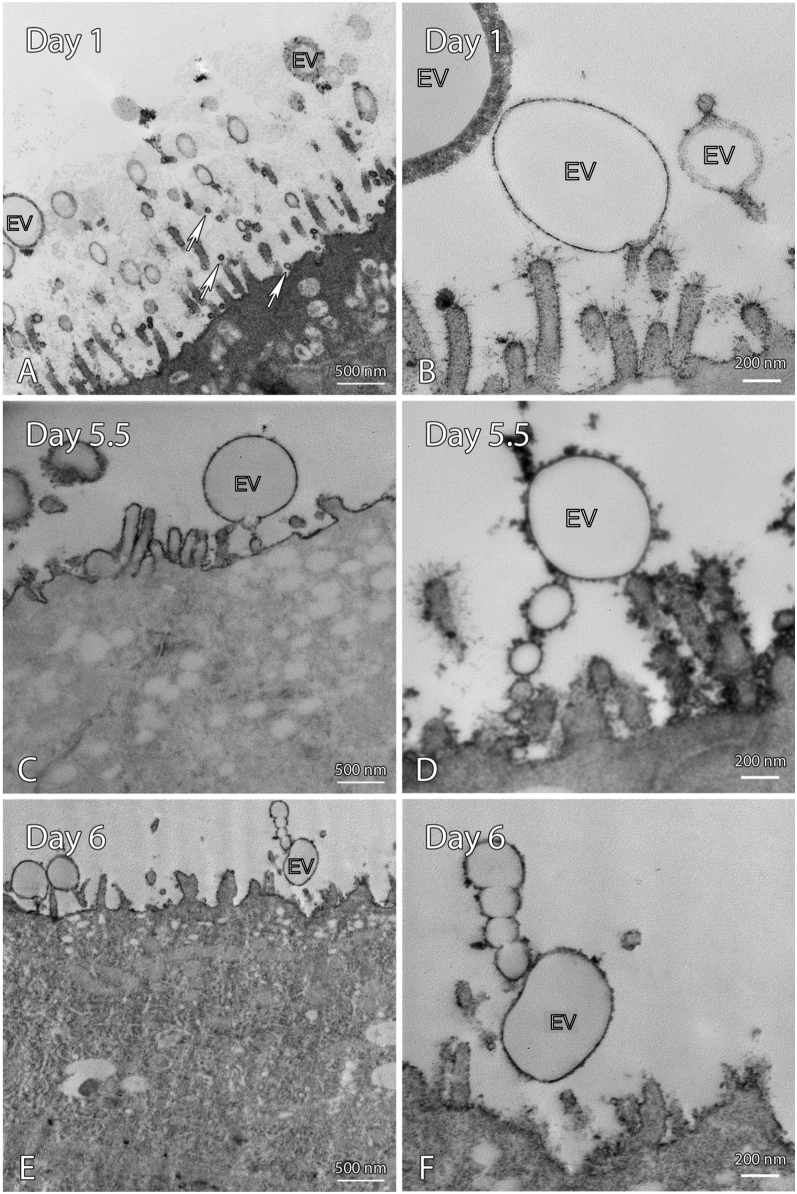
TEM of EVs in UECs on day 1 (A and B), 5.5 (C and D) and 6 (E and F) of early pregnancy. EVs of various sizes were present in the luminal space of the uterus on all days of early pregnancy studied. Scale bars A, C, E 500 nm and B, D, F are 200 nm. (UECs, uterine epithelial cells; EV, extracellular vesicles; arrow = small extracellular vesicles).

**Figure 2:**
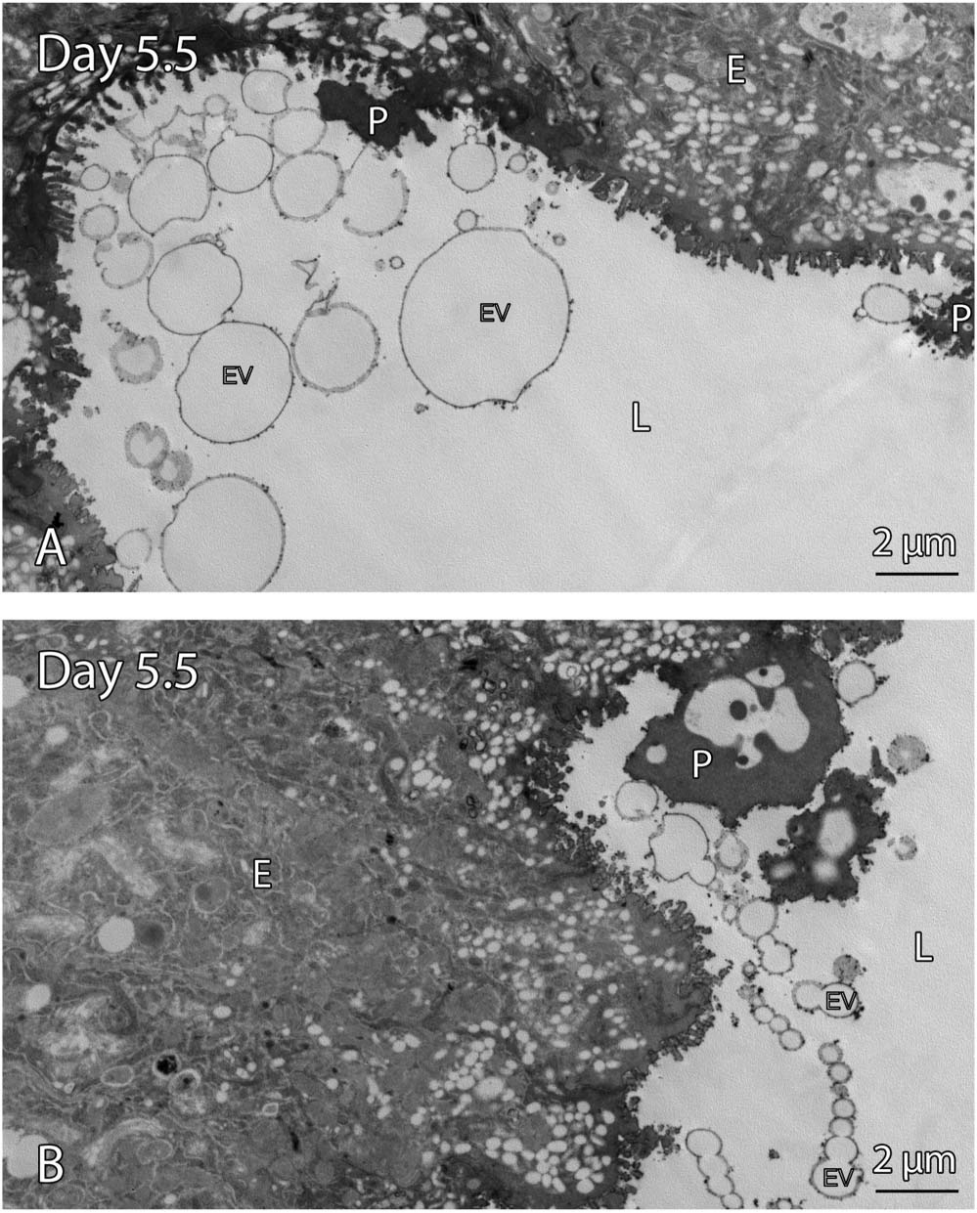
TEM of EVs in the uterine lumen on day 5.5 (A and B) of early pregnancy. A large number of EVs with various sizes and membrane thickness are seen in the luminal space on day 5.5 of pregnancy. Some EVs are near pinopods and appear to aggregate and accumulate together. All scale bars are 2 μm. (EV, extracellular vesicles; L, lumen; E, epithelium; P, pinopods).

EVs are present on day 1, 5.5 and day 6 of pregnancy in the luminal cavity of the uterus when examined by TEM (Figs 1 and 2). EVs of varying sizes and membrane thicknesses were observed. On day 5.5 of pregnancy, numerous EVs were observed in the uterine lumen, some associated with pinopods (Fig. 2). Numerous EVs were seen to cluster and fuse together regardless of size and membrane composition on all days of pregnancy studied (Figs 1 and 2).

### Syntaxin 2 is present in UECs during early pregnancy

**Figure 3:**
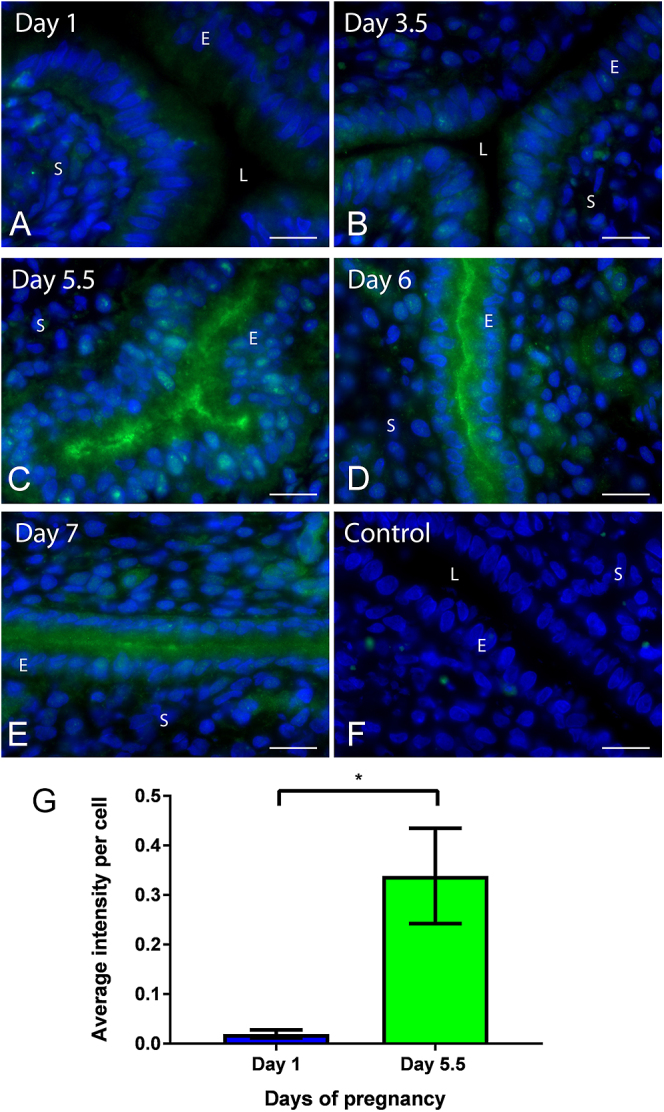
Syntaxin 2 localisation in rat uterus during early pregnancy. Day 1 (A) and 3.5 (B) of pregnancy show diffuse cytoplasmic localisation of syntaxin 2 in UECs. Day 5.5 (C) and 6 (D) of pregnancy syntaxin 2 is concentrated in the apical region of UECs. On day 7 (E) of pregnancy, syntaxin 2 is found cytoplasmically with some apical staining. Non-immune control (F) shows no staining in UECs. Unpaired two-tailed Student’s *t*-test (G) found that syntaxin 2 had a significantly greater intensity per cell on day 5.5 compared to day 1 of pregnancy in UECs. (*P < 0.05) *n* = 5; error bar is the mean ± S.E.M. All scale bars are 20 μm. (L, lumen; E, epithelium; S, stroma).

Indirect immunofluorescence labelling and fluorescence microscopy revealed that syntaxin 2 is present in UECs during early pregnancy in the rat uterus (Fig. 3). On day 1, 3.5 and 7 of pregnancy, syntaxin 2 is found cytoplasmically throughout the cell, with a small amount of apical staining seen on day 7 (Fig. 3A, B, E). On day 5.5 and 6, the time of receptivity, syntaxin 2 is localised in the cytoplasm and apically in UECs (Fig. 3C and D). Non-immune controls performed with all immunofluorescence protocols showed no staining in UECs. A representative image of day 5.5 non-immune controls is shown (Fig. 3F). Measurements of fluorescence intensity in image data taken from days 1 and 5.5 found significantly more syntaxin 2 per epithelial cell on day 5.5 compared to day 1 of pregnancy; *P < 0.05; *n* = 5 (Fig. 3G).

### SNAP23 is present in UECs during early pregnancy

**Figure 4:**
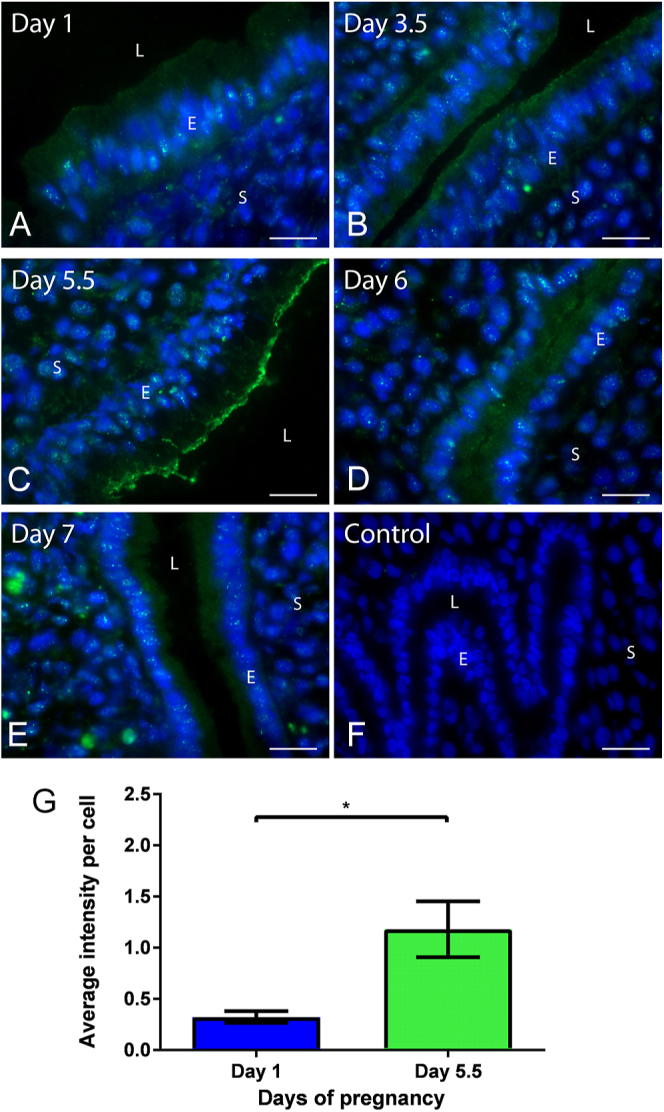
SNAP23 localisation in rat uterus during early pregnancy. Day 1 (A) and 3.5 (B) of pregnancy show diffuse cytoplasmic localisation of SNAP23 in UECs. Day 5.5 (C) of pregnancy SNAP23 is concentrated in the apical region of UECs. Day 6 (D) and 7 (E) of pregnancy SNAP23 are cytoplasmic. Non-immune control (F) shows no staining in UECs. Unpaired two-tailed Student’s *t*-test (G) found that SNAP23 had a significantly greater intensity per cell on day 5.5 compared to day 1 of pregnancy in UECs. (*P < 0.05) *n* = 5; error bar is the mean ± S.E.M. All scale bars are 20 μm. (L, lumen; E, epithelium; S, stroma).

SNAP23 is present in UECs during early pregnancy in the rat uterus when examined through indirect immunofluorescence microscopy (Fig. 4). On days 1, 3.5, 6 and 7, SNAP23 is localised throughout the cytoplasm in UECs (Fig. 4A, B, D, E). On day 5.5, however, SNAP23 is localised apically (Fig. 4C). Non-immune controls were performed with all immunofluorescence protocols, showing no staining in UECs. A representative image of day 5.5 non-immune controls is shown (Fig. 4F). Fluorescent intensity measurements found significantly more SNAP23 staining per epithelial cell on day 5.5 compared to day 1 of pregnancy; *P < 0.05; *n* = 5 (Fig. 4G).

### Western blot analysis of syntaxin 2 and SNAP23

**Figure 5:**
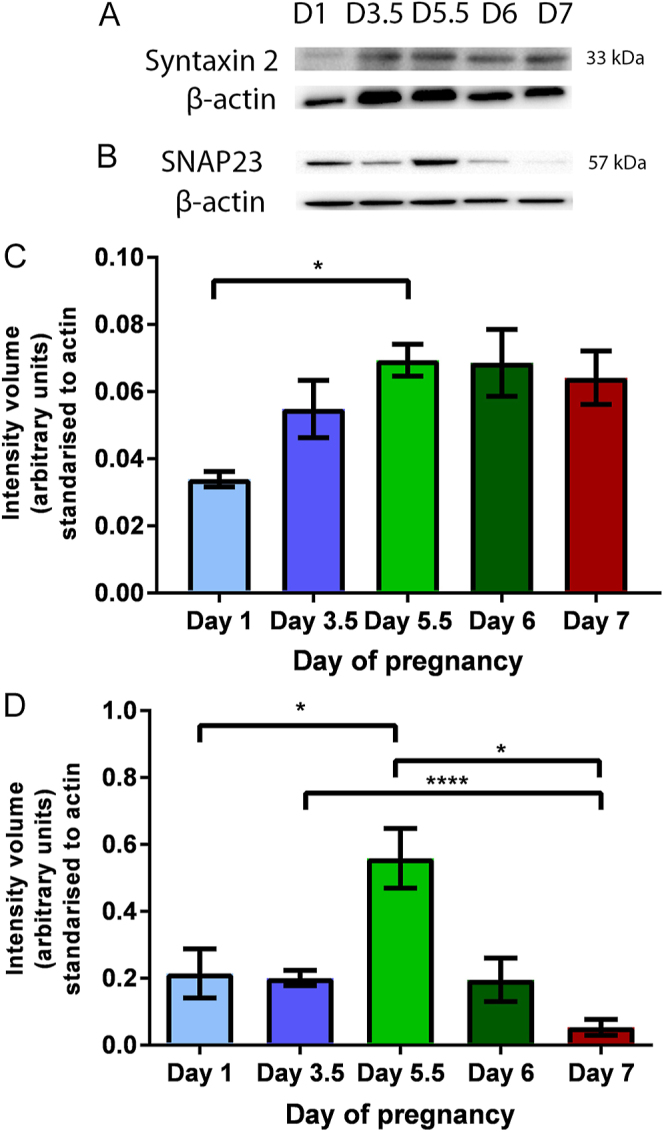
Western blot analysis of syntaxin 2 and SNAP23 in isolated UECs. Syntaxin 2 is present at 33 kDa in isolated UECs during day 1, 3.5, 5.5, 6 and 7 of pregnancy. (A) SNAP23 is present at 57 kDa in isolated UECs during day 1, 3.5, 5.5, 6 and 7 of pregnancy. (B) Blots seen are representative of *n* = 5 blots used for the analysis. (A and B) β-actin was used as a loading control. Densitometric and statistical analysis (one-way ANOVA) found a significant increase in syntaxin 2 on day 5.5 compared to day 1 (*P < 0.05) *n* = 5. (C) Densitometric and statistical analysis (one-way ANOVA) found a significant increase in SNAP23 on day 5.5 compared to day 1. SNAP23 also showed a significant decrease on day 7 compared to day 3.5 and 5.5 (*P < 0.05, ***P < 0.0001) *n* = 5. (D) Error bar is the mean ± SEM.

Western blot analysis on the predicted 33 kDa molecular weight band shows syntaxin 2 in UECs on all days of early pregnancy examined and that the amount of syntaxin 2 was significantly higher on day 5.5 compared to day 1 (Fig. 5A and C). Similarly, SNAP23 (57 kDa) is present in UECs on all days of early pregnancy (Fig. 5B), but that SNAP23 was significantly higher on day 5.5 compared to day 1 and 7. A significant decrease in SNAP23 was also observed between day 3.5 and 7 (Fig. 5D).

### SNAP23 is present in uterine luminal secretion at day 5.5 of pregnancy

**Figure 6:**
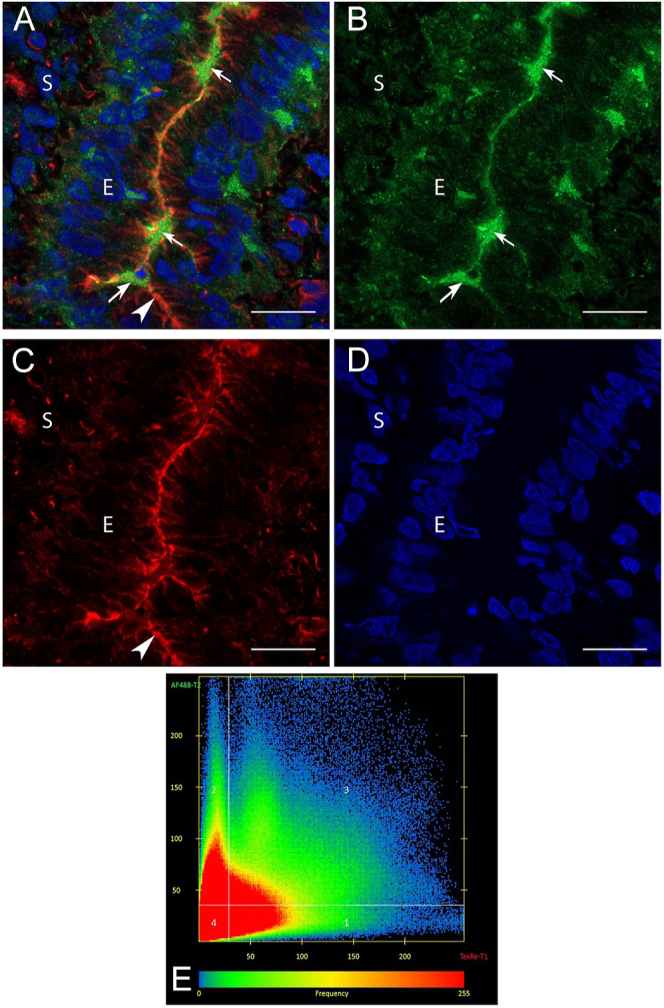
Confocal microscopy-triple labelling of SNAP23 and phalloidin and co-localisation coefficient in UECs on day 5.5 of pregnancy. Merged channels showing the localisation of phalloidin, SNAP23 and nuclei in UECs on day 5.5 of pregnancy. (A) Green channel shows that SNAP23 (arrow) is present in secretions within the luminal space, outside the UECs. (B) Red channel shows that phalloidin (arrowhead) localises apically in UECs. (C) Blue channel shows that the nuclei of UECs. (D) Pearson co-localisation coefficient (PCC) scatter plot (E) showing SNAP23 does not co-localise with phalloidin, with an average PCC of 0.117, *n* = 5. All scale bars are 20 μm. (L, lumen; E, epithelium; S, stroma).

**Figure 7:**
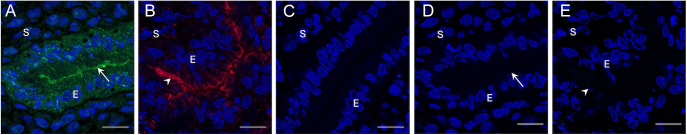
Confocal microscopy control images. All images were taken with red, green and blue channels and merged. SNAP23 staining (arrow) shows that there is no cross talk (bleed through) from the red channel. (A and D) Phalloidin staining (arrowhead) shows that there is no cross talk (bleed through) from the green channel. (B and E) Non-immune control shows no staining in UECs. (C) All scale bars are 20 μm. (L, lumen; E, epithelium; S, stroma).

Co-localisation experiments between SNAP23 and phalloidin in UECs on day 5.5 revealed no apparent co-localisation of SNAP23 and actin (Fig. 6). UECs on day 5.5 of pregnancy demonstrated phalloidin (actin) staining apically and at the apico-lateral junctional areas in UECs, representing the location of the actin cytoskeleton and apical border of UECs (Fig. 6A and C). SNAP23 staining was localised in the entire cavity of the luminal space on day 5.5 (Fig. 6A and B). PCC was calculated between SNAP23 and phalloidin with an average PCC of 0.117; *n* = 5 (Fig. 6E). SNAP23-only (Fig. 7A and D) and phalloidin-only (Fig. 7B and E) control was also imaged with all three filters and there was no cross talk or bleed through between the channels. Non-immune controls were conducted as described above and exhibited no staining (Fig. 7C). The phalloidin marker was incompatible with the fixative used for syntaxin 2 staining, so similar co-localisation was unable to be performed for this protein.

## Discussion

This study is the first to detect SNAP23 in the uterine lumen during the time of receptivity in the rat. This investigation also detected EVs in the uterine lumen during the period of early pregnancy. Syntaxin 2 and SNAP23, both involved in the control of exocytosis, were found to be increased in UECs at the time of uterine receptivity in the rat. We suggest that SNAP23, syntaxin 2 and EVs collectively contribute to the microenvironment for blastocyst implantation.

SNAP23 is found in UECs at 57 kDa on all days of early pregnancy and not at 23 kDa, as previously documented (Zhu *et al.* 2017). It is suggested that SNAP23 (23 kDa) form a heterodimer with syntaxin 2 (34 kDa), resulting in a 57 kDa band. This higher band has been observed in other studies where syntaxin 2 and SNAP23 can form a heterodimer by interacting with VAMP2 to form a SNARE complex (Chen *et al.* 2000, Hu *et al.* 2002, Chintagari *et al.* 2006). VAMP2 is in UECs during early pregnancy and is significantly higher at the time of receptivity (Kalam *et al.* 2020). This heterodimer, also referred to as an acceptor SNARE complex, allows continued cycling of SNARE components for additional fusion events, making exocytosis more efficient as it is more kinetically favourable (Low *et al.* 1998, Hong 2005, Jahn & Scheller 2006). The physical bond for SNAP and syntaxin heterodimers can be so strong that they are toxin- and SDS-resistant. Therefore, it is expected that in highly polarised UECs, as seen *in vivo*, SNAP23 exists as a heterodimer or a pre-assembled complex that plays a major role in the timely apical exocytosis responsible for maintaining the optimal microenvironment for blastocyst implantation.

Cellular actin is known to interact with SNAP23 to facilitate exocytosis (Lang *et al.* 2000, Pendleton & Koffer 2001, Eitzen 2003). Thus, this study performed co-localisation analysis of SNAP23 and actin in UECs and found that actin did not interact with SNAP23. Instead, SNAP23 was found in the luminal space of the receptive uterus, which is an interesting finding. While SNAP 23 is well-known to be associated with intracellular vesicles (Low *et al.* 1998), it is less commonly found in luminal secretions. There is some evidence that SNARE proteins including SNAP23 is found in enriched fractions of EV (Lischnig *et al.* 2022, Welsh *et al.* 2024), which may be the case in our rat early pregnancy model. The appearance of SNAP23 in ULF at the time of uterine receptivity may prove to be a significant clinical finding as a potential predictor of receptivity; however, further studies are necessary.

Syntaxin 2 and SNAP23 are seen in the apical region of UECs at the time of uterine receptivity in this study. However, co-localisation experiments of syntaxin 2 and SNAP23 or actin were not possible due to the different protocols for both antibodies, so the potential overlap in their distribution or release of syntaxin 2 into the uterine lumen could not be confirmed. Syntaxin 2 in the apical region of UECs on day 5.5, 6 and 7 suggests a role in exocytosis during blastocyst implantation. Previous studies have found syntaxin 2 in the following epithelial secretory cells: pancreatic acinar cells, parotid acinar cells and alveolar type II cells, where it is involved in vesicular release (Chen *et al.* 1999, Imai *et al.* 2003, Abonyo *et al.* 2004, Pickett *et al.* 2005). The secretion in the uterine lumen during receptivity is unique and is produced for a finite period to create a microenvironment to assist implantation (Ng *et al.* 2013, Altmäe *et al.* 2017, Liang *et al.* 2017, Zhang *et al.* 2017). Our results indicate that, in UECs, syntaxin 2 is likely facilitating apical vesicular release and may promote exocytosis to allow release of vesicle content.

The EVs seen in the uterine lumen during early pregnancy are consistent with other species including mice (Griffiths *et al.* 2008) and humans (Hart *et al.* 2022). Previous studies have shown that conditioned media from *in vitro* EEC cells led to an increase in sperm capacitation, and hence, fertilisation capacity (Griffiths *et al.* 2008, Franchi *et al.* 2016). The EVs observed on day 1 in the present study may also be facilitating sperm capacitation.

There is equally as much information on the role of EVs during uterine receptivity (Das & Kale 2020, Sui *et al.* 2023), especially in maternal–foetal communication with the EVs containing a range of lipids, proteins and RNAs, providing a protected mechanism of transport. A study in porcine uterine fluid showed that EVs containing proteins including MEP1B increase trophoblast proliferation and migration and thus improve blastocyst implantation rates (Hong *et al.* 2023). A recent study found an alteration in the contents of EVs, particularly miRs, of the uterine lumen in rats after ovarian hyperstimulation (Huang *et al.* 2024), suggesting this as a mechanism for the known decrease in blastocyst implantation following ovarian hyperstimulation (Nicholson *et al.* 2010, Lindsay & Murphy 2014, Lindsay *et al.* 2016).

Another aspect of uterine receptivity is the change in proteins in the apical plasma membrane of UECs including the appearance of LIF (Namiki *et al.* 2023) and integrin α5β3 (Aplin 1996), both of which are essential for successful blastocyst implantation. The change in protein composition of the apical plasma membrane of UECs may involve the movement of EVs seen in the apical cytoplasm of receptive UECs (Parr 1982), together with the apical localisation of SNARE proteins – SNAP23, syntaxin-2 in this study and VAMP2 (Kalam *et al.* 2020) to allow docking of these vesicles with the apical target membrane and subsequent incorporation of these proteins into the apical membrane.

The appearance of large EVs in the uterine luminal space during early pregnancy and significant increase in SNARE proteins and the apical localisation at the time of uterine receptivity suggests a role for SNAP23 and syntaxin 2 in the regulation of the apical membrane composition and contribution to the ULF contents and composition. These changes are known to be essential to uterine receptivity and the successful implantation of the blastocyst during early pregnancy and may in part be mediated by control of the release of EVs from these UECs.

This study found EVs in the uterine luminal space during early pregnancy and significant increase in SNARE proteins syntaxin 2 and SNAP23 in the apical region of UECs, contributing to the receptive change that leads to uterine receptivity. Overall, these findings show that SNAP23, syntaxin 2 and EVs may be collectively working together to create a favourable microenvironment for blastocyst implantation.

## Supplementary materials



## Declaration of interest

The authors declare that there is no conflict of interest that could be perceived as prejudicing the impartiality of the work reported.

## Funding

Financial support was provided by the Australian Research Council, the Ann Macintosh Foundation of the Discipline of Anatomy and Histology, Commercial Development and Industry Partnership Fund (CDIP Fund) USYD and the Murphy Laboratory.

## Author contribution statement

S N K designed the study, performed all experiments, analysed data and took the lead in writing the manuscript. All authors provided critical feedback and helped shape the research, analysis and manuscript.
