# Supplementary material for: Extracellular vesicles, syntaxin 2 and SNAP23 in the uterine microenvironment of the rat
Source: Reproduction. 2025 May 29;169(6):e240188. doi: 10.1530/REP-24-0188 (PMC12125629; doi:10.1530/REP-24-0188)
Supplement: Supplementary file 1 [file supplementary_materials.pdf]

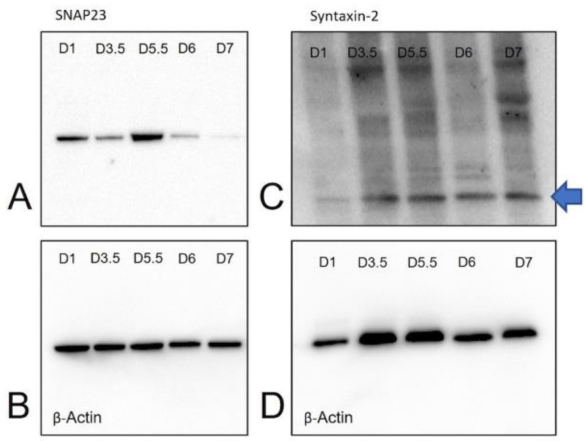

Supplementary Figure 1: Blot transparency for SNAP23 and Syntaxin 2

(A) Full, uncropped blot for SNAP23 at 57kDa and its corresponding β-actin blot (B). (C) Full, uncropped blot for Syntaxin 2 at 33 kDa (blue arrow) and corresponding β-actin blot (D).
